# The Influence of Pregnancy on Sweet Taste Perception and Plaque Acidogenicity

**DOI:** 10.1007/s10995-016-2199-2

**Published:** 2016-12-28

**Authors:** H. Sonbul, H. Ashi, E. Aljahdali, G. Campus, P. Lingström

**Affiliations:** 1 Department of Operative Dentistry, Faculty of Dentistry, King Abdulaziz University, Jeddah, Saudi Arabia; 2 Department of Preventive Dentistry, Faculty of Dentistry, King Abdulaziz University, Jeddah, Saudi Arabia; 3 Department of Cariology, Institute of Odontology, Sahlgrenska Academy, University of Gothenburg, Box 450, Gothenburg, Sweden; 4 Department of Obstetrics and Gynecology, Faculty of Medicine, King Abdulaziz University, Jeddah, Saudi Arabia; 5 Department of Surgery, Microsurgery and Medicine, School of Dentistry, University of Sassari, Sassari, Italy; 6 WHO Collaborating Centre for Epidemiology and Preventive Dentistry, Milan, Italy

**Keywords:** Dental caries, Plaque pH, Pregnancy, Saudi Arabia, Taste perception

## Abstract

*Objectives* Women undergo different physiological and oral changes during pregnancy and this may increase the risk of dental caries and other oral diseases. The aim of the present study was to investigate changes in biofilm acidogenicity and correlate them to sweet taste perception in pregnant and non-pregnant women. *Methods* Three groups of Saudi women participated in this cross-sectional study: (1) women in early pregnancy (n = 40/mean age 29.6 years/DMFT 10.7), (2) women in late pregnancy (n = 40/29.5 years/DMFT 10.8) and (3) non-pregnant women (n = 41/27.7 years/DMFT 12.3). Changes in plaque pH were determined by using colour-coded indicator strips before and after a 1-min rinse with a 10% sucrose solution. A taste perception test determining sweet preference and threshold levels was also performed. *Results* A significant difference regarding plaque pH was seen between the early, late and non-pregnant women when calculated as the area under the curve (*p* < 0.05). Regarding the taste perception tests, taste preference and threshold were correlated (*p* < 0.001, *r* = 0.6). Between the three groups, a statistically significant difference was seen in taste threshold and taste preference respectively (*p* = 0.001 and *p* < 0.001). *Conclusions* The findings in this study suggest that pregnant women may undergo taste changes and experience lower plaque pH, which may result in an increased risk of dental caries.

## Significance

*What is already known on this subject?* It’s well known that women undergo several changes when pregnant which may affect their predisposition to medical and oral diseases. However, the exact cause for oral health changes is still not clear and assumptions have been made about different etiological factors. *What this study adds to this subject?* This study adds new information about oral health in pregnant women and how this may increase the risk for dental caries. More in detail, the plaque acidogenicity and how it differs between pregnant and non-pregnant women is in focus.

## Introduction

Pregnant women experience several changes during pregnancy. These modifications are not limited to systemic, physiological and hormonal changes. They include alterations in the oral cavity that make pregnant women more prone to oral infections (Barak et al. 2003). Specific preventive measures and treatments are needed during pregnancy to avoid oral infections (Meyer et al. 2014). These oral changes through pregnancy might be caused by different factors such as the changes in estrogen and progesterone hormones, a lower immune response and oral bacterial changes (Silk et al. 2008).

It is known that pregnant women might be more susceptible to developing dental caries and have been found to have a higher prevalence of dental caries (Martinez-Beneyto et al. 2011; Vergnes et al. 2012). It has been suggested that dietary changes occurring in pregnancy, such as an increase in the consumption of carbohydrates, affect the susceptibility of pregnant women to dental caries (Russell and Mayberry 2008). An increased craving for sweets and fast foods has been found among pregnant women in a recent study (Orloff et al. 2016). In addition, other oral factors such as increased acidity in the mouth/saliva and a reduction in saliva production may also have an impact (Russell and Mayberry 2008). A decrease in saliva production is usually accompanied by a decrease in plaque pH and an increase in the retention of dietary carbohydrates on the tooth surface (Lingström and Birkhed 1993). The pH of saliva has been found to be lower in pregnant women compared with non-pregnant women (Rockenbach et al. 2006), but no studies of plaque acidogenicity in relation to pregnancy have been performed.

In relation to changes in dietary habits, it is also important to consider smell and taste perception. It has also been suggested that pregnant women may dislike the taste of toothpaste and oral mouth-rinse products (Martinez-Beneyto et al. 2011). The changes are known to occur most frequently during the early part of the pregnancy, after which they decline by the end of pregnancy and usually disappear after delivery (Brown and Toma 1986; Nordin et al. 2004).

Other factors which may influence oral health are related to the pregnant women’s beliefs and attitudes, such as a lack of dental check-ups and treatment (Ressler-Maerlender et al. 2005). In addition, pregnant women might fear some of the dental treatments and their effect on pregnancy outcome (Ressler-Maerlender et al. 2005). A lack of dental visits during pregnancy has been found among women who do not seek dental treatment prior to pregnancy (Boggess et al. 2010). Pregnant women may also refrain from the appropriate oral hygiene measures due to acid reflux, nausea and vomiting (Martinez-Beneyto et al. 2011; Vergnes et al. 2012).

All the above-mentioned factors may increase the risk of oral infections and their consequences in pregnancy. An early assessment of oral diseases such as dental caries and periodontal diseases and dietary recommendations are therefore important (Silk et al. 2008). In spite of our knowledge of pregnant women and the effect of pregnancy on general and oral health, there is still a lack of knowledge regarding the correlation.

The aim of the study was to compare the changes in sweet taste perception and plaque pH between pregnant and non-pregnant women, including comparisons between early and late pregnancy. This aim is based on the hypothesis that taste changes in pregnancy, which may in turn influence the taste preference for sweet intake and dietary pattern and consequently increase the risk of caries. The null hypothesis was that no difference would be found between pregnant and non-pregnant women and the two pregnant groups, in terms of sweet taste preference and plaque acidogenicity.

## Methods

### Administrative Procedure

A detailed study plan was submitted to the responsible institute in Jeddah, Saudi Arabia. In addition, ethical approval was obtained from the local ethics committee at King Abdulaziz University, Jeddah, Saudi Arabia (#003–12) and informed consent was obtained from each volunteer. All the participants were informed about the aims and procedure of the study before they signed the consent form. Before starting the study, all the participants were given a code that was used for the further handling of all data and they were reassured about the confidentiality of the data collected from them.

### Participants and Study Design

The study had a cross-sectional design and the participants were chosen randomly, using a pre-randomised list, from the Obstetric and Gynecology Clinic, King Abdulaziz University Hospital, Jeddah, Saudi Arabia. Every other subject (nos. 1, 3, 5 etc) was included and put into the respective group.

The study comprised a total of 121 subjects, 80 pregnant and 41 non-pregnant women (control). The pregnant group was divided into two subgroups; early (1–20 weeks in pregnancy) and late (21–40 weeks in pregnancy) pregnant women. Sample size calculation was based on results from previous pH measurements with an estimated difference in pH-fall of 0.4, SD 0.5, significance level of 5 and 80% power. The subjects had similar socio-economic status (SES), free of medical diseases, not taking any medication other than prenatal vitamins and with a minimum of 20 teeth present. The SES was determined by two questions concerning educational level and yearly income. The subjects came to the Obstetric and Gynecology Clinic for examination after refraining from eating, drinking, toothbrushing including use of mouthwash and smoking during the last hour prior to the test and not using any antibiotics during the last prior month.

The following tests were performed in this order: (1) saliva assessment, (2) plaque acidogenicity, (3) sweet taste perception test and (4) caries registration. Prior to the clinical examination, demographic data on factors such as age, education and occupation were obtained verbally from the participants. Two dentists participated in the data collection; each individual collected one set of data, i.e. all tests of one kind.

### Saliva Assessment

Unstimulated and stimulated saliva samples were collected. For unstimulated saliva, the subjects were seated in a relaxed position while saliva was drooled passively into the vial. Stimulated saliva samples were collected by getting the subjects to chew on a piece of paraffin wax while saliva was collected. The unstimulated and stimulated secretion rates were calculated in mL/min.

A microbiological assessment of mutans streptoccoci and lactobacilli was performed using a chairside test (CRT^®^ bacteria, Ivoclar-Vivadent, Schaan, Liechtenstein). The dip slide was covered in saliva after which bacteria were grown in an incubator for 48 h at 37 °C. The results were compared with a chart provided by the manufacturer and scored from 0 to 3 corresponding to number of colony-forming units per mL saliva. Buffer capacity was assessed by soaking a buffer strip (CRT Buffer, Ivoclar-Vivadent) in the collected saliva. After 5 min, the strip was compared to the chart provided by the manufacturer in order to determine the buffer capacity level as low, medium or high.

### Plaque Acidogenicity

The plaque acidogenicity was assessed using the “strip method” (Carlen et al. 2010). A pH indicator strip (Spezialindikator, Merck, Darmstadt, Germany) was used to measure the pH value (4.0–7.0). Each strip was cut into three pieces and inserted in the interproximal area (under the contact point of the teeth) of the premolar/molar in the left and right upper region, before (0 min) and 2, 5, 10 and 20 min after a 1-min mouth rinse with 10 mL of a 10% sucrose solution. In order to assess the pH values, comparisons were made between the colour appearing on the inserted strip and the index provided by the manufacturer.

### Sweet Perception Test

The sweet taste threshold (i.e. the lowest concentration at which the subject identified the presence of sucrose and distinguish it from water) and taste preference (i.e. the solution that was recorded as their sweet taste preference level.) were tested using a modified version of the one used by Furquim et al. (2010). Each subject tasted ten different sucrose solutions with concentrations ranging from 1.62 to 821.52 gm/L. They were served as 10 mL quantities in a plastic medicine cup.

Before and between tasting each concentration, the subjects actively rinsed their mouths with 10 mL of filtered deionised water and waited for 2 min between each tasting trial. When tasting the solutions, the subjects were instructed to use a sufficient amount of the solution given to guarantee exposing all the taste buds in the oral cavity for at least 5 s before they were allowed to spit out the solution.

The solutions were tested in order of increasing concentration and the subjects were asked to identify their taste threshold level and secondly, they were asked to indicate the preferred solution.

### Caries Registration

The subjects were examined for the number of decayed, missed and filled teeth (DMFT) with the exclusion of unerupted teeth, congenitally missing teeth or supernumerary teeth, teeth removed for reasons other than dental caries and primary teeth retained in the permanent dentition. A DMFT of 28 was the maximum, meaning that all the teeth were affected. Third molars were excluded from the calculation.

### Statistical Analysis

The mean, standard deviation and range for variables were calculated using the IMB^®^ SPSS^®^ (PASW version 21.0 IBM^®^ Chicago, IL, USA). The independent *t* test was used to determine the differences between non-pregnant and pregnant groups. The pregnant group was divided into two subgroups depending on how far their pregnancy had progressed-early pregnant and late pregnant. The difference between the three groups (non-pregnant, early pregnant and late pregnant) was tested by one-way ANOVA. Difference in proportion of microbiological data and groups were tested by Chi square test. For plaque pH, the area under the curve below pH 5.7 (AUC_5.7_) and pH 6.2 (AUC_6.2_) was calculated using a special computer program. The relationship between variables was tested using Pearson’s correlation. A *p* value of <0.05 was considered statistically significant. Multinomial regression analysis was run to evaluate the impact of the different variables recorded on the two different groups (non-pregnant and pregnant) and the three different groups (non-pregnant, early pregnant, late pregnant).

## Results

The mean age, number of teeth and DMFT for the three groups are shown in Table 1. No statistically significant differences were found when comparing any of the groups for the number of teeth and DMFT value at baseline. Additional analyses showed no significant differences when comparing the three components (D, M, F) between the three groups.

**Table 1:** Mean ± SD and range for age, DMFT and saliva analysis (non-stimulated and stimulated salivary secretion rate and number of lactobacilli and mutans streptococci) for pregnant, non-pregnant, early pregnant and late pregnant women

	Pregnant (n = 80)		Non-pregnant (n = 41)		Early pregnant n=(40)		Late pregnant (n = 40)		*p* value	*p* value
	Mean ± SD	Range	Mean ± SD	Range	Mean ± SD	Range	Mean ± SD	Range		
Age	29.5 ± 6.0	18–43	27.7 ± 8.8	16–54	29.6 ± 6.8	18–43	29.5 ± 5.2	18–39	0.227^a^	0.395^b^
Number of teeth	24.2 ± 3.7	14–28	22.7 ± 4.7	10–28	24.5 ± 3.2	18–28	24.0 ± 4.1	14–28	0.072^a^	0.127^b^
DMFT	10.7 ± 5.2	0–24	12.3 ± 5.3	0–23	10.7 ± 5.0	2–23	10.8 ± 5.5	0–24	0.108^a^	0.277^b^
Unstimulated saliva (mL/min)	0.5 ± 0.3	0.0–3.2	0.6 ± 0.6	0.1–3.2	0.5 ± 0.4	0.1–1.4	0.4 ± 2.7	0.0–1.4	0.104^a^	0.143^b^
Stimulated saliva (mL/min)	1.7 ± 1.1	0.2–6.0	1.8 ± 0.9	0.0–2.5	1.5 ± 0.8	0.3–3.8	1.9 ± 1.3	0.6–6.0	0.734^a^	0.219^b^
Lactobacilli count (score 0/1/2/3)	5/28/33/14	0.0–3.0	5/12/19/5	0.0–3.0	1/14/18/7	0.0–3.0	4/14/15/7	0.0–3.0	0.02^c^	0.55^d^
Mutans streptococci (score 0/1/2/3)	5/21/42/12	0.0–3.0	2/15/19/5	0.0–3.0	1/11/23/5	0.0–3.0	4/10/19/7	0.0–3.0	0.03^c^	0.24^d^

^a^Statistically significant difference between non-pregnant and pregnant groups (independent *t* test)

^b^Statistically significant difference between non-pregnant, early pregnant and late pregnant groups (ANOVA)

^c^Statistically significant difference between non-pregnant and pregnant groups (Chi square test)

^d^Statistically significant difference between non-pregnant, early pregnant and late pregnant groups (Chi square test)

### Saliva Assessment

As shown in Table 1, small numerical variations in the unstimulated saliva secretion rate ranging from 0.4 to 0.6 mL/min were found between the different groups, with the highest value for the non-pregnant women (ns). The corresponding values for the stimulated saliva secretion rate ranged between 1.5 and 1.9 mL/min (ns). The pregnant women were found to have higher numbers of mutans streptococci and lactobacilli than the non-pregnant ones (*p* < 0.05) (Table 1). The majority of the non-pregnant women (56%) showed a high saliva buffering capacity, while nearly half the pregnant women (46%) showed a medium buffering capacity (*p* < 0.01; data not shown). A positive correlation was found between saliva buffering capacity and AUC_5.7_ and AUC_6.2_ respectively (*p* < 0.01, *r* = 0.244; *p* < 0.05, *r* = 0.21).

### Plaque pH

**Fig. 1:**
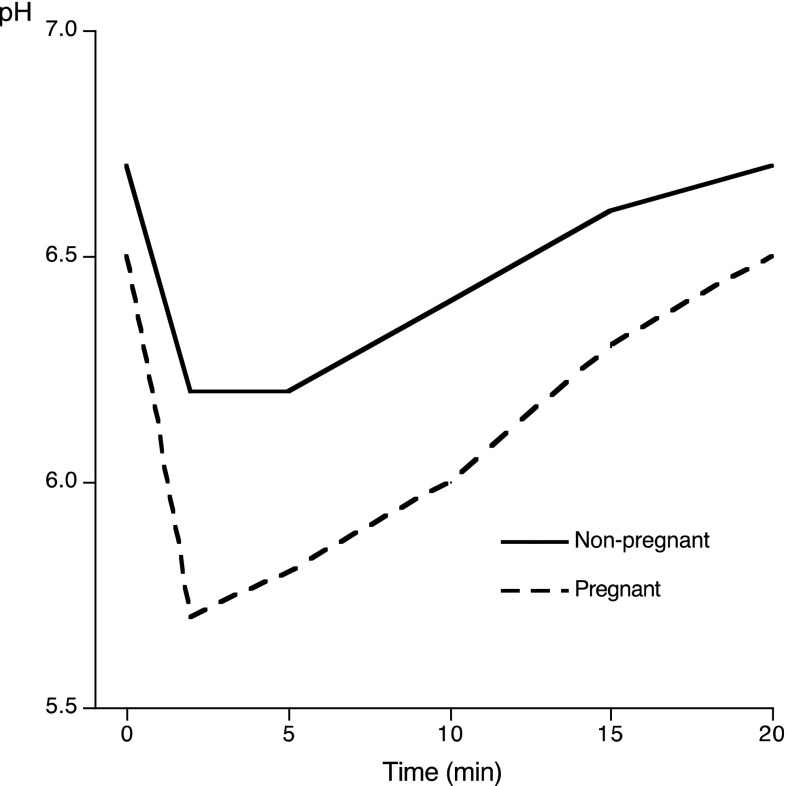
Changes in plaque-pH after a mouthrinse with 10 mL 10% sucrose in the non-pregnant and pregnant women

**Fig. 2:**
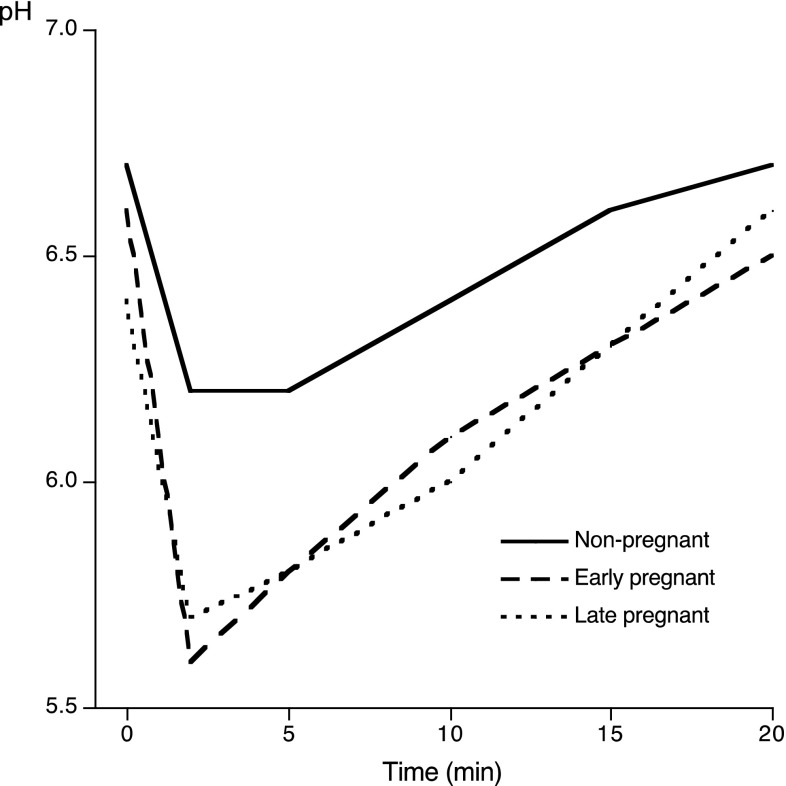
Changes in plaque-pH after a mouthrinse with 10 mL 10% sucrose in the non-pregnant, early pregnant and late pregnant women

The most pronounced pH fall at the different time points was seen for the pregnant women (*p* < 0.05; *p* < 0.01; *p* < 0.001) compared with the non-pregnant ones (Table 2; Fig. 1). When comparing the two pregnant groups, a statistically significant difference was only found at baseline (*p* < 0.05) (Fig. 2). The mean minimum pH value was 5.5 in the pregnant women and 5.9 in the non-pregnant women, with a wider range for the pregnant women of 4.0–6.8 (Table 2). When it came to the maximum pH fall, the most pronounced value was found in the early pregnancy group (1.2 pH units). In terms of the area under the curve (AUC) for pH values of 6.2 and 5.7, the largest areas were seen when viewing the pH value in the pregnant group (5.8 respectively 2.1) in comparison with the non-pregnant group (2.5 and 0.6 respectively); both these values were statistically significant (*p* < 0.01). A statistically significant difference between the early, late pregnant and non-pregnant women was found for the area under the curve at pH 6.2 (*p* < 0.05).

**Table 2:** Mean ± SD and range for plaque pH (pH at the different time points, minimum pH, maximum pH-fall, AUC_6.2_ and AUC_5.7_ for pregnant, non-pregnant, early pregnant and late pregnant women)

	Pregnant (n = 80)		Non-pregnant (n = 41)		Early pregnant (n = 40)		Late pregnant (n = 40)		*p* value^a^	*p* value^b^
	Mean ± SD	Range	Mean ± SD	Range	Mean ± SD	Range	Mean ± SD	Range		
Plaque pH										
0 min	6.5 ± 0.6	4.4–7.0	6.7 ± 0.4	4.9–7.0	6.6 ± 0.5	5.3–7.0	6.4 ± 0.6	4.4–7.0	0.014	0.004
2 min	5.7 ± 0.6	4.5–7.0	6.2 ± 0.5	5.2–7.0	5.6 ± 0.6	4.6–6.8	5.7 ± 0.6	4.5–6.8	0.000	<0.01
5 min	5.8 ± 0.5	4.4–7.0	6.2 ± 0.5	5.1–7.0	5.8 ± 0.5	4.7–6.8	5.8–0.5	4.4–6.5	0.000	<0.01
10 min	6.0 ± 0.6	4.0–7.0	6.4 ± 0.5	6.3–7.0	6.1 ± 0.7	4.0–7.0	6.0 ± 0.5	4.6–7.0	0.003	0.014
15 min	6.3 ± 0.6	4.6–7.0	6.6 ± 0.5	5.3–7.0	6.3 ± 0.6	4.9–7.0	6.3 ± 0.6	4.6–7.0	0.013	0.044
20 min	6.5 ± 0.5	4.9–7.0	6.7 ± 0.5	5.3–7.0	6.5 ± 0.6	4.9–7.0	6.6 ± 0.5	4.9–7.0	0.171	0.378
Minimum pH	5.5 ± 0.5	4.0–6.8	5.9 ± 0.5	4.9–6.8	5.5 ± 0.5	4.0–5.5	5.6 ± 0.5	4.4–6.5	0.000	0.001
Maximum pH fall	1.0 ± 0.5	0.0–2.5	0.8 ± 0.4	0.0–1.7	1.2 ± 0.7	0.4–2.5	0.8 ± 0.5	0.0–1.8	0.220	0.001
AUC_6.2_	5.8 ± 7.2	0.0–29.6	2.5 ± 4.3	0.0–16.9	6.1 ± 7.3	0.0–27.7	5.6 ± 7.1	0.0–29.6	0.002	0.025
AUC_5.7_	2.1 ± 4.3	0.0–19.7	0.6 ± 1.6	0.0–7.3	2.3 ± 4.4	0.0–17.7	2.0 ± 4.2	0.0–19.7	0.004	0.077

^a^Statistically significant difference between non-pregnant and pregnant groups (independent *t* test)

^b^Statistically significant difference between non-pregnant, early pregnant and late pregnant groups (ANOVA)

A strong correlation was found between AUC_5.7_ and AUC_6.2_ (*p* < 0.01, *r* = 0.940). Both AUC values were highly correlated to the minimum pH (*p* < 0.01, *r* = −0.704 and *p* < 0.01, *r* = −0.823 respectively). A significant correlation was found between lactobacilli and each of the following variables; minimum pH, AUC_6.2_ and AUC_5.7_ respectively (*p* < 0.05; *r* = −0.188, *p* < 0.05; *r* = 0.208, *p* < 0.05; *r* = 0.187).

### Taste Examination

When it came to the taste threshold, a statistically significant difference was found when comparing pregnant and non-pregnant women (*p* < 0.05), with a higher value among the pregnant women (Table 3). The early pregnancy group showed the highest mean value for taste threshold (24.1 gm/L), followed by late pregnancy (14.1 gm/L) and finally the non-pregnant group (12.5 gm/L) (*p* = 0.001). Fifty-five per cent of the early pregnancy group reported a taste threshold for a sucrose solution concentration of 25.67 gm/L and higher compared with 30.0% for late pregnancy and 17.1% for the non-pregnant women (Table 4). The higher concentration reported for taste threshold was a sucrose solution concentration of 102.70 gm/L, which was only observed in the early pregnancy group (Table 4).

**Table 3:** Mean ± SD, median and range for taste threshold and taste preference data for pregnant, non-pregnant, early pregnant and late pregnant women

	Pregnant (n = 80)		Non pregnant (n = 41)		Early pregnant n=(40)		Late pregnant (n = 40)		*p* value^a^	*p* value^b^
	Mean ± SD	Range	Mean ± SD	Range	Mean ± SD	Range	Mean ± SD	Range		
Taste threshold	19.1 ± 16.9	1.6–102.7	12.5 ± 11.4	1.6–51.3	24.1 ± 20.6	1.6–102.7	14.1 ± 10.4	1.6–51.3	0.013	0.001
Taste preference	77.7 ± 84.5	6.5–410.8	45.9 ± 38.4	6.5–205.3	108.6 ± 106.0	6.5–410.8	46.9 ± 35.6	6.5–102.7	0.005	0.000

^a^Statistically significant differences between non–pregnant and pregnant groups (independent *t* test)

^b^Statistically significant differences between non-pregnant, early pregnant and late pregnant groups (ANOVA)

**Table 4:** Frequency and percentage n (%) of taste threshold and taste preference for the different sucrose solutions chosen by pregnant, non-pregnant, early pregnant and late pregnant women

Sucrose solution (gm/L)	Pregnant (n = 80)		Non-pregnant n=(41)		Early pregnant n=(40)		Late pregnant n=(40)	
	Taste threshold n (%)	Taste preference n (%)	Taste threshold n (%)	Taste preference n (%)	Taste threshold n (%)	Taste preference n (%)	Taste threshold n (%)	Taste preference n (%)
1.625	5 (6.3)	0 (0)	4 (9.8)	0 (0)	3 (7.5)	0 (0)	2 (5.0)	0 (0)
3.251	6 (7.5)	0 (0)	6 (14.6)	0 (0)	3 (7.5)	0 (0)	3 (7.5)	0 (0)
6.50	16 (20.0)	3 (3.8)	8 (19.5)	3 (7.3)	4 (10.0)	1 (2.5)	12 (30.0)	2 (5.0)
12.83	19 (23.8)	13 (16.3)	16 (39.0)	9 (22.0)	8 (20.0)	4 (10.0)	11 (27.5)	9 (22.5)
25.67	25 (31.3)	13 (16.3)	5 (12.2)	6 (14.6)	14 (35.0)	3 (7.5)	11 (27.5)	10 (25.0)
51.34	8 (10.0)	21 (26.3)	2 (4.9)	17 (41.5)	7 (17.5)	12 (30.0)	1 (2.5)	9 (22.5)
102.70	1 (1.3)	21 (26.3)	0 (0)	5 (12.2)	1 (2.5)	11 (27.5)	0 (0)	10 (25.0)
205.38	0 (0)	6 (7.5)	0 (0)	1 (2.4)	0 (0)	6 (15.0)	0 (0)	0 (0)
410.76	0 (0)	3 (3.8)	0 (0)	0 (0)	0 (0)	3 (7.5)	0 (0)	0 (0)
821.52	0 (0)	0 (0)	0 (0)	0 (0)	0 (0)	0 (0)	0 (0)	0 (0)

A comparison between pregnant and non-pregnant women revealed a statistically significant difference for taste preference (*p* < 0.01) (Table 3). The highest preferred sucrose solution, 410.76 gm/L, was found for the early pregnant women, followed by the late pregnant and the non-pregnant women (Table 4). The sucrose solution concentration of 102.70 gm/L was preferred by 50.0% of the early pregnant women, 25.0% of the late pregnant and 14.6% of the non-pregnant women (*p* < 0.001).

Taste threshold and taste preference were found to be highly correlated (*p* < 0.001, *r* = 0.6). No correlation was found when comparing taste preference or taste threshold with any of the pH variables (ns).

In pregnancy, buffer capacity, AUC_6.2_, DMFT and taste threshold showed a statistically positive RRR with respect to non-pregnancy (Table 5). In early pregnancy, the buffer capacity (RRR = 2.49) and AUC_6.2_ (RRR = 1.13) were statistically significantly different with respect to the base outcome (non-pregnancy) (Table 6). During late pregnancy, several variables (buffer capacity, AUC_6.2_, DMFT, taste threshold) showed a statistically positive RRR with respect to non-pregnant women.

**Table 5:** Multinomial regression outcomes using as a dependent variable for the two groups (non pregnant and pregnant)

Variables	RRR	Std. Err.	p > |z|	(95% conf. interval)
Buffer capacity	2.00	0.99	0.001	1.56–5.74
AUC_6.2_	1.14	0.06	0.014	1.03–1.26
DMFT	0.62	−2.05	0.040	0.39–0.98
Taste threshold	2.10	0.70	0.030	1.09–4.02

Number of obs = 121; p < 0.001; log likelihood = −60.87

**Table 6:** Multinomial regression outcomes using as dependent variable for the three groups (non pregnant, early pregnant, late pregnant)

Variables	RRR	Std. Err.	p > |z|	(95% conf. interval)
Early pregnant				
Buffer capacity	2.49	0.87	0.01	1.26–4.86
AUC_6.2_	1.13	0.06	0.02	1.02–1.26
DMFT	0.67	0.16	0.09	0.39–1.07
Taste threshold	1.72	0.62	0.13	0.85–8.56
Late pregnant				
Buffer capacity	4.00	1.55	<0.01	1.87–8.56
AUC_6.2_	1.14	0.06	0.02	1.03–1.27
DMFT	0.58	0.16	0.04	0.34–0.98
Taste threshold	2.77	1.09	<0.01	1.28–6.00

Number of obs = 121; p < 0.01; log likelihood = − 113.78

## Discussion

The aim of this study was to evaluate sweet taste perception and plaque pH during pregnancy. The first null hypothesis was rejected, as it was found that pregnant women experienced more pronounced plaque acidogenicity, which may also affect the caries risk. The second hypothesis was also rejected, i.e. that women experience changes in taste, in particular in favour of sweetness. This may lead to changes in dietary habits, resulting in particular in an increased craving for sweets, which could in turn increase the risk of dental caries.

The taste threshold and taste preference test was slightly modified from the method used by Furquim et al. (2010), which was in turn based on studies by (Nilsson and Holm 1983; Zengo and Mandel 1972). After pilot testing prior to start, it was decided that the taste threshold and taste preference would be tested at the same time. The main finding was that both the sweet taste threshold and preference level were higher in the pregnant group, showing a higher sucrose concentration threshold/preference than the non-pregnant group.

It has previously been shown that taste during pregnancy start to change in the first trimester (Brown and Toma 1986; Nordin et al. 2004). Furthermore, other studies have reported a noticeable preference for sweets in the second trimester (Belzer et al. 2010; Bowen 1992). This is confirmed by the present study in which more than half the early pregnant women chose the solutions of ≥25.67 gm/L when tested for their sweet taste threshold and they were also the only group that chose the highest recorded sucrose concentration (102.70 gm/L). The same finding applied when it came to the sweet taste preference, with half the early pregnant group preferring the highest reported solution.

The study revealed a difference in sweet taste preference between women in early and late pregnancy, with a higher preference among the early group. This indicates that the risk of dietary changes is greatest in the early stage of pregnancy. Special attention should therefore be paid to the dietary habits of early pregnant women in particular and recommendations should be made to avoid food with a high sugar content that could increase the risk of caries. The exact explanation for this increasing preference for sweet tastes in pregnant women is still unknown. However, it has been suggested that it is caused by changes in taste and smell or metabolic changes during pregnancy (Hook 1978). Other possible explanations mentioned in the literature are the mother’s need to increase her food intake to be able to provide nutrients to the growing baby and the effect of fluctuations in sex hormones on food intake (Faas et al. 2010).

In terms of plaque acidogenicity, pregnant women displayed a higher pH fall and a larger area under the curve (AUC_5.7_ and AUC_6.2_) compared with the non-pregnant ones. This higher, prolonged pH fall may increase the risk of solubility for the tooth hard tissue (Aranibar Quiroz et al. 2014). Both enamel and dentine are then at higher risk for demineralization at pH <5.7 respective pH <6.2. In addition, an interesting finding was the lower resting pH among the pregnant group compared with the non-pregnant one. These changes in plaque pH during pregnancy have not been evaluated in previous studies. The increased threshold and preference level for sweets may lead to a change in sweet dietary intake, which has been reflected in the presence of a higher number of cariogenic micro-organisms. A lower salivary pH in pregnant women has previously been reported (Rockenbach et al. 2006), but this could not be confirmed in the present study.

The remineralisation capability in pregnant women might be affected. This could be explained by biologically related factors such as the variation in plaque composition and salivary composition or behavioural changes; in particular, oral hygiene habits (Russell and Mayberry 2008). Dental caries was not the outcome variable in this study, but a recent study from our group has shown that the minimum plaque-pH value is strongly correlated to the number of initial carious lesions (Aranibar Quiroz et al. 2014). The lower pH found among the pregnant women thus indicates that they should be regarded as a high caries risk group that requires special attention and caries-preventive measures.

This study did not reveal any association between taste preference/threshold and any of the plaque-pH variables. This could have been expected, as dietary intake is known to be strongly correlated to biofilm character and acidogenicity (Aranibar Quiroz et al. 2003). However, even if a difference in taste perception was found in the present study, no evaluation was made of whether this had led to actual changes in the dietary habits of pregnant women. Furthermore, it is well known that Saudi individuals as a whole have a fairly high intake of sweets (Bakhotmah 2011). A study conducted by Collison et al. (2010) on Saudi children suggested that, with advancing age, there is a tendency towards food with high sugar content.

This study indicates that pregnant women should be treated as high-risk individuals for dental caries. Dietary advice should therefore focus on reducing the frequency of sugar consumption or ensuring that sugar is consumed with meals and not between meals. It is also important to emphasise the importance of preventive measures including optimal fluoride use. Even if prenatal practitioners feel that pregnancy increases the risk of dental caries, only 20% refer pregnant women for general dental check-ups (Boutigny et al. 2016). Dental health professionals should collaborate with prenatal and antenatal care to educate pregnant women on the normal biological oral changes that may occur during pregnancy and guide them towards consuming less sugar and sweets, as well as stressing the importance of adopting the appropriate oral hygiene habits and techniques such as toothbrushing twice daily with fluoridated tooth paste, flossing and making regular dental visits. Furthermore, even if pregnancy is limited in time, it still has to be seen as a risk factor in a wider perspective. For several women, this condition may be repeated over a longer time period. In addition, it is well known that the change in dietary habits may persist in mothers of newborn babies. Studies reveal that oral health during pregnancy not only affects the pregnant women but also has an impact on the oral health of newborn babies and subsequently on adolescents (Boggess and Edelstein 2006; Meyer et al. 2014). An oral health care and preventive program for pregnant woman will therefore not only be beneficial to the mother but will also have a positive impact on the oral health of the child.

Due to the difficulty involved in identifying women before pregnancy and assigning them in a longitudinal study, a cross-sectional study design was used. A group of non-pregnant women from the same socio-economic level was included as controls. All the women took part in all the examinations. However, it has previously been found that pregnant women may find sitting in a dental chair uncomfortable and prolonged sitting can cause pressure on the vena cava and lead to supine hypotensive syndrome (Barak et al. 2003). A few women in the present study reported an unpleasant feeling from the sucrose solution with an experience of gagging reflex. However, all the women managed to perform all the tests according to the instructions they were given.

## Conclusion

Findings suggest that pregnant women may undergo taste changes, which may result in an increased risk of dental caries, which is accompanied by an increased plaque acidogenicity. For future studies, it would therefore be interesting to investigate oral changes, including factors affecting caries and the dietary habits of pregnant women and mothers of newborn babies, longitudinally. It is therefore suggested that pregnant women should be re-examined after the birth of their babies to create a longitudinal design.
